# Chitinases of *Bacillus thuringiensis*: Phylogeny, Modular Structure, and Applied Potentials

**DOI:** 10.3389/fmicb.2019.03032

**Published:** 2020-01-14

**Authors:** Sheila A. Martínez-Zavala, Uriel E. Barboza-Pérez, Gustavo Hernández-Guzmán, Dennis K. Bideshi, José E. Barboza-Corona

**Affiliations:** ^1^Graduate Program in Biosciences, Life Science Division, University of Guanajuato Campus Irapuato-Salamanca, Guanajuato, Mexico; ^2^School of Biological Sciences, The University of Edinburgh, Edinburgh, United Kingdom; ^3^Department of Biological Sciences, California Baptist University, Riverside, CA, United States; ^4^Department of Entomology, University of California, Riverside, Riverside, CA, United States; ^5^Food Department, Life Science Division, University of Guanajuato Campus Irapuato-Salamanca, Guanajuato, Mexico

**Keywords:** *Bacillus thuringiensis*, chitinases, domains analysis, regulation, phylogeny, insecticidal activity, antifungal, nematocidal

## Abstract

The most important bioinsecticide used worldwide is *Bacillus thuringiensis* and its hallmark is a rich variety of insecticidal Cry protein, many of which have been genetically engineered for expression in transgenic crops. Over the past 20 years, the discovery of other insecticidal proteins and metabolites synthesized by *B. thuringiensis*, including chitinases, antimicrobial peptides, vegetative insecticidal proteins (VIP), and siderophores, has expanded the applied value of this bacterium for use as an antibacterial, fungicidal, and nematicidal resource. These properties allow us to view *B. thuringiensis* not only as an entity for the production of a particular metabolite, but also as a multifaceted microbial factory. In particular, chitinases of *B. thuringiensis* are secreted enzymes that hydrolyze chitin, an abundant molecule in the biosphere, second only to cellulose. The observation that chitinases increase the insecticidal activity of Cry proteins has stimulated further study of these enzymes produced by *B. thuringiensis*. Here, we provide a review of a subset of our knowledge of *B. thuringiensis* chitinases as it relates to their phylogenetic relationships, regulation of expression, biotechnological potential for controlling entomopathogens, fungi, and nematodes, and their use in generating chitin-derived oligosaccharides (ChOGs) that possess antibacterial activities against a number of clinically significant bacterial pathogens. Recent advances in the structural organization of these enzymes are also discussed, as are our perspective for future studies.

## Introduction

**FIGURE 1:**
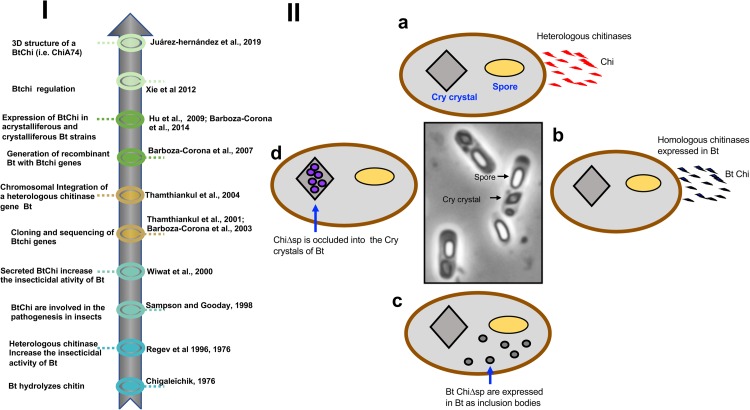
**(I)** Timeline. A brief history of the use of homologous and heterologous chitinases to increase the insecticidal activity of *B. thuringiensis*. **(II)** Different options for the development of recombinant strains of *B. thuringiensis* expressing chitinases. (a) The bacterium can be transformed with heterologous chitinase genes obtained from bacteria other than *B. thuringiensis*. Chitinases harbor signal peptides and are secreted from the cells. (b) The bacterium can be transformed with homologous chitinase genes obtained from different strains of *B. thuringiensis*. Chitinases harbor signal peptides and are secreted from the cells. (c) Chitinase genes from *B. thuringiensis* are engineered and the signal peptide is deleted. Chitinases are expressed inside the cells as inclusion bodies along with the spores and insecticidal Cry proteins. (d) Chitinase genes lacking signal peptides are transcriptionally fused to the C-terminal encoding moiety of *cry1* genes, facilitating the formation of disulfide bridges between the chimeric construct and Cry proteins. This strategy putatively allows the formation of chimeric crystals made of Cry and chitinases. Bt, *B. thuringiensis*; Chi, chitinases; Bt Chi, chitinases from Bt, Cry crystals, parasporal bodies made of Cry proteins; ChiΔsp, chitinase lacking signal peptide; Bt ChiΔsp, chitinase from Bt lacking a signal peptide.

The sporogenic Gram-positive bacterium *Bacillus thuringiensis* has gained preeminence among microbial bioinsecticides, including those based on formulations of entomopathogenic fungi, viruses, nematodes, and other bacteria, owing to its insecticidal properties and commercial success worldwide (∼211,000,000 USD; Lacey et al., 2015). The insecticidal activities of *B. thuringiensis* are generally target-specific and result from proteinaceous protoxins (Cry, crystal; Cyt, cytolytic; δ-endotoxins) that are synthesized at high levels and then crystallized during the sporulation phase of growth (Figure 1). These crystalline inclusions (Cry crystals), also called parasporal bodies, are easily observed with phase contrast microscopy. Cry crystals represent the hallmark of *B. thuringiensis* and its presence distinguishes it from other *Bacillus* species, including *B. cereus*, *B. anthracis*, and *B. subtilis*. When Cry crystals are ingested, they dissolve in the midgut of susceptible larvae. Subsequently, solubilized Cry protoxins are proteolytically activated by enzymes in the digestive juice to generate active entomotoxins that bind to various receptors in the midgut, a precursor to destabilization of osmotic balance and degradation of the midgut epithelia and larval mortality. Indeed, larvae of a number of agronomical pests (e.g., *Plutella xylostella*, *Spodoptera frugiperda*) and arthropod vectors of human diseases (e.g., *Culex*, *Aedes*, and *Anopheles* species) are susceptible to these toxins. Moreover, a wide variety of *cry* genes have been cloned and modified for expression in transgenic plants to resist attack and infestation by insects (Palma et al., 2014). Most studies on *B. thuringiensis* have focused on molecular characterization of Cry proteins, mechanisms of toxicity, crystal structure, and identification of new strains that have commercial potential. In contrast, significantly less attention has been paid to other biomolecules of applied interest. Nevertheless, there is growing interest in less-known metabolites synthesized by *B. thuringiensis*, including chitinases (Chi), bacteriocins, vegetative insecticidal proteins (VIP), and siderophores, as they have potential applied value not only in insect control, but also as antibacterial, fungicidal, nematicidal and acaricidal agents, and their ability to directly or indirectly promote plant growth (Lee et al., 2009; De la Fuente-Salcido et al., 2013; Casados-Vázquez et al., 2017, 2018; Jouzani et al., 2017; Azizoglu, 2019). In addition, *B. thuringiensis* also synthesizes proteins called parasporins that lack insecticidal activity, but which are active against human cancer cells (Ohba et al., 2009). Interestingly, it was recently reported that Cry1Ab and Cry1Ac are also cytotoxic to cervical cancer (HeLa) cells (Mendoza-Almanza et al., 2019).

Chitin is a polymer composed of repeating units of *N*-acetyl-*D*-glucosamine and it is found in insects, fungi, shrimp shells, fish scales, mollusks, and also in bird plumage and butterfly wings (Morganti, 2012). It is the second most abundant biopolymer in nature, surpassed only by cellulose. Chitosan is a deacetylated chitin derivate with antibacterial activities. It is commonly used in the manufacture of cosmetics, wood dressings, pool-water treatment agents, and edible films. The use of chitin/chitosan present in seafood shells has also been proposed as a viable alternative to plastics which when used indiscriminately present a major environmental hazard (Drahl, 2019). Chitin occurs as highly ordered, crystalline microfibrils, forming three polymorphs (α, β and γ) composed of ∼100 to 8000 GlcNAc residues depending on the chitin source (Kaya et al., 2017). The α-type is the most stable form. It possesses strong intermolecular bonding and is found in shells of crustaceans, fungi cell walls, and in insect cuticles. β-chitin has weak intermolecular interactions, and it has been observed in association with proteins in squid pens, cell walls of diatoms, and in the lorica built by seaweeds or protozoa. γ-chitin is considered to be a combination of α- and β-chitin, and it has been detected in *Ptinus* beetle cocoon fibers, the cocoon of *Orgyia dubia*, and the stomach of *Loligo* sp. (squid) (Herth et al., 2011; Kaya et al., 2017).

Biological structures made of chitin are prime targets for chitinolytic enzymes, and as such, phytopathogenic fungi or pest insects are susceptible to degradation by chitinases (Morales de la Vega et al., 2006; Juárez-Hernández et al., 2015; Hollensteiner et al., 2017). Chitinases are produced and secreted by viruses, prokaryotes, and eukaryotes, including humans, plants, fungi, and insects, which interestingly may or not have chitin. Bacteria synthesize a myriad of chitinolytic enzymes to transform chitin to carbon and nitrogen sources which together with other metabolites promote plant growth. Previously, chitinases were classified as endochitinases, exochitinases (chitobiosidases, chitobiases), and N-acetylglucosaminidases, according to the products generated during the hydrolysis process. Currently, and according to the CAZy database^1^, chitinolytic enzymes are classified in two general groups: chitinases (EC 3.2.1.14) and β-*N*-acetylhexosaminidases (EC 3.2.1.52), previously called endochitinases and exochitinases, respectively. Chitinases cleave chitin chains at internal sites randomly, whereas β-*N*-acetylhexosaminidases hydrolyze chitin from the non-reducing end of the molecule by removing GlcNAc residues. These enzymes are further grouped into different glycoside hydrolases (GH) families based on their amino acid sequence similarities: endochitinases represent four groups (GH18, GH19, GH23, and GH48), whereas β-*N*-acetylhexosaminidases represent six groups (GH3, GH5, GH18, GH20, GH84, GH116). Most bacterial chitinases belong to the GH18 family.

In particular, interest in the applied value of *B. thuringiensis* chitinases (Bt Chi) was initiated in the 1970s when it was demonstrated that enzymes secreted by this bacterium hydrolyzed chitin (Chigalel̆chik, 1976). Later, it was shown that *B. thuringiensis* produces chitinases that when used in combination with other components, including Cry proteins, contributed to its virulence (Smirnoff, 1974; Regev et al., 1996; Guttmann and Ellar, 2000). Soon thereafter, cloning of genes coding for *B. thuringiensis* chitinases was reported (Thamthiankul et al., 2001; Barboza-Corona et al., 2003) which initiated the development of recombinant *B. thuringiensis* strains expressing homologous chitinases. More recently, elucidation of the three-dimensional structure of chitinase ChiA74 (Juárez-Hernández et al., 2019) laid a foundation for performing directed evolutionary studies to create a cassette of more stable and efficient enzymes for practical purposes. Several excellent reviews have been published on Cry proteins and a few other metabolites of *B. thuringiensis* (De la Fuente-Salcido et al., 2013; Palma et al., 2014; Jouzani et al., 2017; Azizoglu, 2019), but not on chitinases produced by this bacterium. In this review, our objective is to survey pertinent information published on chitinases of *B. thuringiensis*, with a focus on their phylogeny, regulation, and potential to control insects, microorganisms and nematodes. Recent advances in the structural organization of these enzymes are also discussed, as are our perspective for future studies.

## Chitinases of *B. thuringiensis* and Its Modular Organization

Chitinases produced by *B. thuringiensis* may play different roles that contribute to the survival of this bacterium under different conditions. For example, these enzymes can be used (i) to sequester and assimilate chitin and use it as a sole source of carbon, and (ii) to act as a virulence factor that promotes the establishment of successful infection by *B. thuringiensis* by compromising structural components, such as the peritrophic membrane through which activated Cry proteins transit in the target host. (iii) Additionally, it is well established that *B. thuringiensis* propagate in moribund and deceased larvae following infection and that several bacterial-based enzymes including proteases, lipases, esterases, and chitinases amplify this process. Chitinases, in particular, can participates in the destruction of the cuticle thereby facilitating the release and dissemination of toxins and spores, and establishment of the bacterium in other ecological niches (e.g., soil, phylloplane) (Argola-Filho and Loguercio, 2014; Malovichko et al., 2019).

Chitinases of *B. thuringiensis* are synthesized at a basal level under the regulation of native promoters, and increased yields require the addition of chitin to the culture medium. To significantly increase chitinase yield for applied commercial purposes, it is essential to identify a myriad of chitinase genes from different niches together with the physical and biochemical parameters that affect the activity of their corresponding enzyme, and also to select the best candidates for hyperexpression in recombinant strains of *B. thuringiensis*. To date, more than 1000 complete genomes and whole-genome shotgun sequences of *B. thuringiensis* have been deposited in the National Center for Biotechnology Information^2^. However, ∼50 chitinase genes harbored in different subspecies of this bacterium have been reported in GenBank. Most of the chitinase genes were cloned and sequenced in China (∼41%), followed by Mexico and Pakistan (∼17 and 15%, respectively), and ∼2–4% from Thailand, Brazil, Tunisia, Turkey, Vietnam, Japan, Korea, Egypt, and India (Table 1). Based on amino acid sequence alignments, Barboza-Corona et al. (2008) classified chitinases of *B. thuringiensis* in four groups. Most of these chitinases showed identities from ∼90 to 99%, but the main difference was observed in the C-terminal end of the deduced amino acid sequences. For example, Group 3 differs from groups 2 and 1 because its members have an addition of ∼12 or 24 amino acids, respectively. Group 1 includes chitinase ChiA74 from *B. thuringiensis* subsp. *kenyae* (Barboza-Corona et al., 2003), whereas four contains only ChiA71, a chitinase from *B. thuringiensis* subsp. *pakistani* (Thamthiankul et al., 2001). ChiA74 and ChiA71 were the first two chitinases from *B. thuringiensis* genes that were cloned, sequenced and characterized. Interestingly, the main difference between ChiA74 (Group 1) and ChiA71 (Group 4), was the presence of 118 amino acids in the former, but absent in the latter. Also, a 93-amino acid sequence present in the C-terminal region of ChiA71 is absent in ChiA74 (Barboza-Corona et al., 2008). As bacterial chitinases are secreted enzymes they also contain a signal peptide, usually located at the N-terminus, to initiate translocation of the enzyme through the cell wall architecture to the external environment (Li and Li, 2009). Interesting, the signal peptide of chitinase of *B. thuringiensis* is also recognized by the secretion machinery of *E. coli*, a Gram-negative bacterium (Barboza-Corona et al., 2003; De la Fuente-Salcido et al., 2016). Most of the chitinases of *B. thuringiensis* have a molecular mass of ∼70 kDa (Thamthiankul et al., 2001; Barboza-Corona et al., 2003, 2008; Zhong et al., 2003; Lin and Xiong, 2004; Driss et al., 2005; De la Fuente-Salcido et al., 2016; Honda et al., 2017), although chitinases of ∼30 kDa have been reported (Arora et al., 2003; Chen et al., 2007); in general, they have optimal pH activity around 6.5–8.5 with a range temperature of 50–60°C. It is difficult to compare the activity of chitinases from *B. thuringiensis* with other bacterial chitinases, mainly because the differences in the substrates used for the assays and the way to express the units of chitinase activity, i.e., there is no a standardized protocol for determining the activity of bacterial chitinases. Nevertheless, using 4MU-(GlcNAc)_3_ as substrate, it has been observed that ChiA74 from *thuringiensis* has a catalytic efficiency of 1.77 s^–1^μM^–1^ which is higher than ChiA1 from *B. circulans* (0.4 s^–1^μM^–1^), but lower than ChiA and ChiB from *Serratia marcescens* with values of 16 and 8.4 s^–1^μM^–1^, respectively (Watanabe et al., 1993; Casados-Vazquez et al., 2015; Oyeleye and Normi, 2018).

**TABLE 1:** Chitinase genes of *Bacillus thuringiensis* reported in the GenBank.

**Number**	**Accession numbers**	**Subspecies of *B. thuringiensis***	**Reporting year in the GenBank**	**Origin country**	**References**^∗∗∗^
1	BTU89796	*pakistani*	1997	Thailand	Thamthiankul et al., 2001
2	AF424979	*kenyae*	2003	México	Barboza-Corona et al., 2003
3	AF526379	*israelensis*,	2003	China	Zhong et al., 2003
4	AY456381	*entomocidus*	2003	China	Unpublished
5	AY452507	*alesti*	2003	China	Lin and Xiong, 2004
6	AY455290	*canadensis*, strain HD224	2003	China	Unpublished
7	EF211999	Nti^∗∗^, strain BT-7216	2004	China	Unpublished
8	AY452506	*toumanoffi*	2004	China	Unpublished
9	AY279975	*aizawai*	2004	Thailand	Unpublished
10	AJ635226	*kurstaki*	2005	Tunisia	Driss et al., 2005
11	AY074882	Nti	2005	China	Unpublished
12	DQ512474	*colmeri* strain 15A3	2006	China	Unpublished
13	DQ993175	*morrisoni*	2006	Turkey	Unpublished
14	AY129671	*sotto*	2006	China	Unpublished
15	EF427670	Nti	2007	China	Unpublished
16	EU030625	Nti	2007	China	Unpublished
17	EF581163	*kurstaki*, strain HD-73	2008	México	Barboza-Corona et al., 2008
18	EU557354	Nti, strain KR 19-22	2008	Korea	Unpublished
19	EU373094	Nti, strain 97243-1	2008	China	Unpublished
20	EU373095	Nti, strain H14	2009	China	Unpublished
21	GQ183830	*konkukian* strain S4	2009	Pakistan	Unpublished
22	GQ183831	*konkukian* strain S4	2011	Pakistan	Unpublished
23	JX474751	*israelensis*, NRRL HD-522	2012	Egypt	Unpublished
24	HQ418219	Nti, strain RN52	2013	México	Rosas-García et al., 2013
25	HQ418218	Nti, strain MR33	2013	México	Rosas-García et al., 2013
26	HQ418217	Nti, strain MR21	2013	México	Rosas-García et al., 2013
27	HQ418216	Nti, strain MR11	2013	México	Rosas-García et al., 2013
28	HQ418215	Nti, strain MR10	2013	México	Rosas-García et al., 2013
29	HE993892	Nti, strain SBS-BT5	2013	Pakistan	Unpublished
30	KJ010822	Nti, strain HTS-S-38	2014	China	Unpublished
31	KJ508093	Nti, strain DLD171	2014	China	Unpublished
32	HF542112°	Nti, strain BUPM173	2014	Tunisia	Unpublished
33	HF542113°	Nti, strain BUPM85	2014	Tunisia	Unpublished
34	HG792452	Nti, strain SBS-BT6	2014	Pakistan	Unpublished
35	HG792451	Nti, strain SBS-BT3	2014	Pakistan	Unpublished
36	HG792449	Nti, strain SBS-BT5	2014	Pakistan	Unpublished
37	KM249886°	Nti, strain BC-7	2014	India	Unpublished
38	KF671757	*tenebrionis*, strain 9602	2015	China	Ni et al., 2015
39	KJ764712	*tenebrionis*, DSM2803	2015	México	De la Fuente-Salcido et al., 2016
40	KJ676691	Nti, strain I555	2015	Brazil	Unpublished
41	EF103273	*colmeri* 15A3	2015	China	Chen et al., 2007
42	HE995800	Nti, strain SBS-Bt5	2015	Pakistan	Unpublished
43	EU734811°	Nti, isolate 66	2016	Egypt	Unpublished
44	GQ899142°	*kurstaki*	2016	India	Unpublished
45	LC194873	*israelensis* ATCC 35646	2017	Japan	Honda et al., 2017
46	MF630994	*kurstaki* strain MSS1.1	2017	Viet Nam	Unpublished
47	EF197878	Nti	2018	China	Unpublished
48	MK313782	Nti, strain HS66	2019	China	Unpublished
49	MK032857	Nti, strain HD-73	2019	China	Unpublished

*°Partial sequences. ^∗∗^Nti: Not indicated. ^∗∗∗^Information according with the GenBank data (www.ncbi.nlm.nih.gov).*

**FIGURE 2:**
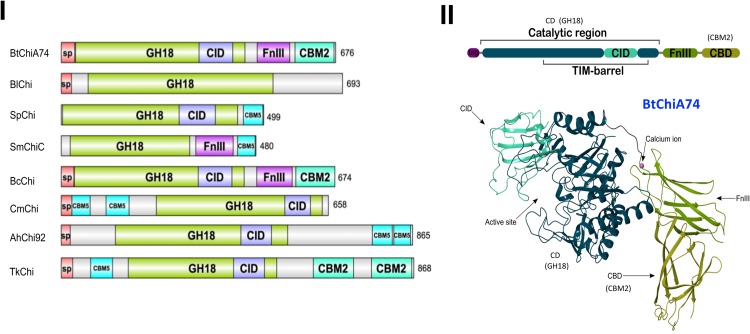
Comparison of the modular structure of chitinases of *B. thuringiensis* with different bacterial chitinases and the three-dimensional structure of the ChiA74 from *B. thuringiensis*. **(I)** Modular alignment between different bacterial chitinases. Protein sequences were analyzed using the Interpro webserver (www.ebi.ac.uk/interpro/beta/) and the figure was built with the program DOG 1.0 (Ren et al., 2009). The nomenclature used in the CAZy database was maintained. Signal peptide, sp; catalytic domains, GH18; CID, chitinase insertion domain; carbohydrate-binding module, CBM; fibronectin type III domain, FnIII. Chitinase (Chi) of: *Bt*, *B. thuringiensis* (accession number AF424979.1); *Bl*, *B. licheniformis* (QAS14701.1); *Sp*, *Serratia proteamaculans* (AGF70636.1); *Sm*, *S. marcescens* (ABI79318.1); *Sm*, *S. marcescens* AHH32576.1; *Bc*, *B. cereus* (FRI-35 AFQ09088.1); *Cm*, *Chitinolyticbacter meiyuanensis* (ATN39892.1); *Ah*, *Aeromonas hydrophila* (AAG09437.1); *Tk*, *Thermococcus kodakarensis* (BAA88380). **(II)** Crystal structure of the chitinase ChiA74 of *B. thuringiensis* with little additions in the nomenclature to match with **(I)**. The catalytic region (CD) corresponds to the GH18, and chitin binding domain (CBD) to the CBM2 showed in panel **(I)**. This clarification is also shown in **(II)**. The three-dimensional structure was first reported by our group in Juárez-Hernández et al. (2019), Scientific Reports, available online at https://doi.org/10.1038/s41598-019-39464-z.

Bacterial chitinases have a modular organization where the catalytic domain can be associated with the following modules: chitinase insertion domain (CID), fibronectin type III-like (FnIII), and chitinase binding domain (CBD) (Juárez-Hernández et al., 2017, 2019). The modular organization can vary, as evidenced by *in silico* comparisons with chitinases from other bacteria using the Interpro webserver^3^. ChiA74 from *B. thuringiensis* has a similar organization with a chitinase from *B. cereus* but differs from other bacterial chitinases (Figure 2). The variation in the modular organization is in agreement with the diversity in chitin substrate structures (Svitil and Kirchman, 1998). Family 18 chitinases composed of enzymes synthesized by *Bacillus* species are divided into subfamilies A and B. These subfamilies differ by the presence of a chitin insertion domain (CID) found in subfamily A, but absent in B. The catalytic domain is composed of a TIM-barrel that contains an (α/β)8-barrel fold found in many different enzymes. This catalytic domain occasionally has a second domain inserted into the active site of the TIM domain, i.e., CID, which participates in binding and catalytic processes (Li and Greene, 2010). *B. thuringiensis* chitinases belong to subfamily A and contain the CID domain (Juárez-Hernández et al., 2019). The CID is composed of five or six anti-parallel β-strand and one α-helix that alongside the TIM barrel substrate-binding cleft forms a wall that increases the depth of the cleft which suggests the CID facilitates orienting and binding longer saccharide substrates (Van Aalten et al., 2000). Most chitinases have one or more chitin-binding domains (CBDs) that are important for interacting with insoluble chitin, and also for facilitating microbial attachment to chitin for subsequent degradation (Wu et al., 2001; Arora et al., 2013; Lobo et al., 2013). The CBDs belong to different types of Carbohydrate-Binding Modules (CBMs) and the location of CBDs in chitinases is variable. For example, chitinase ChiA74 from *B. thuringiensis* has one CBD at the C-terminus (Juárez-Hernández et al., 2017), the chitinase from *Chitinolyticbacter meiyuanensis* (*Cm*Chi1) has two CBDs between the signal peptide and the catalytic domain (Zhang et al., 2018), and chitinase Chi92 from *A. hydrophila* (*Ah*Chi92) contains three repeat CBD domains at the C-terminus (Wu et al., 2001). There is evidence to suggest that the CBD affects how the enzyme accesses glycosidic bonds within chitin strands (Svitil and Kirchman, 1998). Aromatic amino acids (W, Y) in the CBD are highly conserved and appear to be essential for hydrophobic binding with the pyranosyl rings of N-acetylglucosamine residues in chitin (Zhong et al., 2015). In particular for ChiA74 the CBD belongs to the carbohydrate-binding type 2 domain subfamily b (CBM2b). This domain has a conserved region of ∼100 amino acids uncommon in chitinases, but present in xylanases (Wertz et al., 2017). The fibronectin type III-like domain (FnIII) and surface layer homology domains (SLH domain) are present in chitinases of the genera *Bacillus*. In particular, *B. thuringiensis* chitinases possess a FnIII domain composed of a β-sandwich with approximately 100 amino acids. This domain was originally identified in the eukaryotic plasma protein fibronectin and it is part of one of three types of internal repeats (FnI-FnII-FnIII) that arrange other domains in space, acting as a structural spacer (Valk et al., 2017). In bacteria, ∼19% of the FnIII domains are found in proteins directly related to carbohydrate metabolism and proteins that contain carbohydrate-binding domains, such as chitinases and cellulases; the location of this domain is between other domains. According to recent reports, in carbohydrate hydrolyzing enzymes the FnIII domain act as a stable linker in multi-domain proteins, as a carbohydrate surface disruption domain, or as a carbohydrate-binding domain. Watanabe et al. (1994) suggested that FnIII may help maintain the optimal distance and orientation between catalytic and CBDs in *Bc*ChiA1. Also, in ChiA74 the FnIII might act as a stable linker between the catalytic regions (CDs) and the CBD (Juárez-Hernández et al., 2019). On the other hand, it has been suggested that the FnIII domain of a chitinase from *B. thuringiensis* subsp. *tenebrionis* might act synergistically with chitin-binding proteins, helping to attach chitin to the enzyme for efficient substrate hydrolysis (De la Fuente-Salcido et al., 2016).

Finally, the first three-dimensional (3D) structure of the chitinase ChiA74 was elucidated. This crystal structure confirmed the multi-domain assembly of ChiA74 is formed by (i) a CD, (ii) a CID, (iii) a FnIII, and (iv) a chitin binding domain (CBD) (Juárez-Hernández et al., 2019). Moreover, this study demonstrated the importance of the catalytic E211 in the CD, as mutants were inactive against a variety of substrates, including colloidal/crystalline chitins, chitosan, and synthetic fluorogenic compounds, and also suggested that the CBD might play a significant role in the hydrolysis of crystalline chitin.

## Phylogenetic Analysis and Chitinase Expression

**FIGURE 3:**
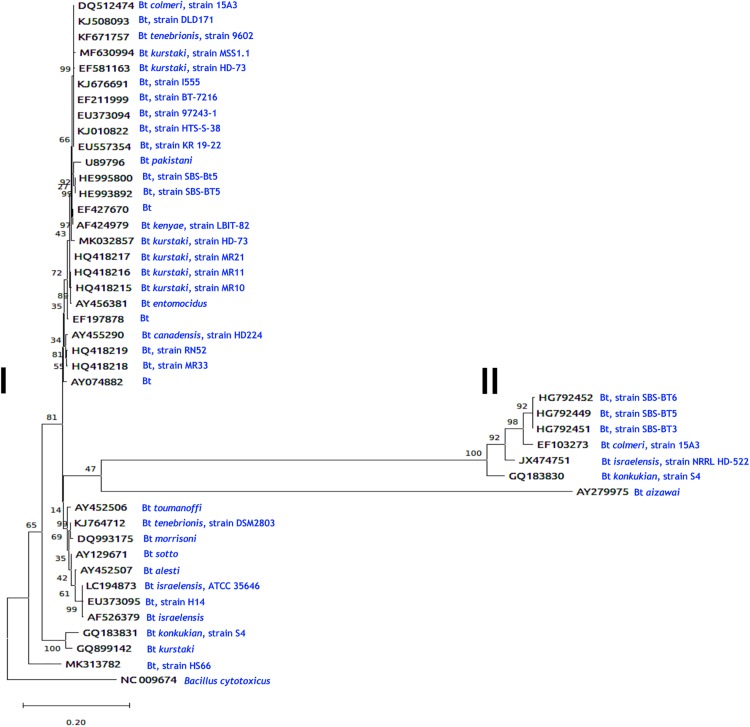
Evolutionary analysis of *Bacillus* chitinases by the Maximum Likelihood method. The evolutionary history was inferred by using the Maximum Likelihood method and the General Time Reversible model (Nei and Kumar, 2000). The tree with the highest log likelihood (-13605.90) is shown. The percentage of trees in which the associated taxa clustered together is shown next to the branches. Initial tree(s) for the heuristic search were obtained automatically by applying Neighbor-Join and BioNJ algorithms to a matrix of pairwise distances estimated using the Maximum Composite Likelihood (MCL) approach and then selecting the topology with superior log likelihood value. A discrete Gamma distribution was used to model evolutionary rate differences among sites [5 categories (+*G*, parameter = 1.7979). The tree is drawn to scale, with branch lengths measured in the number of substitutions per site. This analysis involved 44 nucleotide sequences. There was a total of 3234 positions in the final dataset. Evolutionary analyses were conducted in MEGA X (Kumar et al., 2018). To elaborate this figure, information of the coding sequences of *Bacillus thuringiensis* chitinases reported to date in the GenBank nucleotide sequence database of the National Center for Biotechnology Information (https://www.ncbi.nlm.nih.gov/) were taken. Two main groups were distinguished, **(I)** and **(II)**. After each accession numbers are indicated the *B. thuringiensis* (Bt) subspecies and/or the strain types (e.g., Bt *colmeri*, strain 15A3; Bt, strain DLD171) (see in blue) were chitinases were obtained. *B. cytotoxicus* was used as an external group. Additional information on the *B. thuringiensis* strains is shown in Table 1.

To determine the genetic relationships of *B. thuringiensis* chitinases, information of the coding sequences reported to date in the GenBank nucleotide sequence database of the National Center for Biotechnology Information^4^ were used (see also Table 1). Deduced amino acid sequences were aligned with MUSCLE (MUltiple Sequence Comparison by Log- Expectation^5^). The chitinase from *B. cytotoxicus* [NC_009674; Bazinet (2017)] was used as an outgroup and the resulting alignment was exported as a Pearson/FastA file and then submitted to Findmodel software^6^ to assess which phylogenetic model optimally described the data. Phylogenetic trees were obtained with MEGA X (Kumar et al., 2018) and constructed using the Maximum Likelihood method and General Time Reversible + G model. The tree (Figure 3) with the highest log likelihood was obtained with 1000 bootstrap replicates (Felsenstein, 1985). The phylogram shows an unrooted topology containing some branches with low bootstrap values, supporting a complex evolutionary history for *B. thuringiensis* chitinases. Most sequences with a small number of amino acid replacements were groups in two sister branches with some polytomy with unresolved nodes. A branch sister of *B. cytotoxicus* grouped all *B. thuringiensis* chitinases. The most populated branch separates two sisters, grouping two *B. thuringiensis* subsp. *pakistani* sequences in a branch with 100% bootstrap, and a branch containing six sister groups, one of them showing the largest number of replacements, and the remaining five sisters showing a small number of replacements per site. Sisters with large amino acid replacements branched in two groups, one composed of five chitinases from *B. thuringiensis* subsp. *pakistani*, *colmeri, israelensis*, and *konkukian* and second with a single chitinase from *B. thuringiensis* subsp. *aizawai*.

Regarding target-specific groupings of chitinases, there is scarce information about the effect of chitinases and associated toxicity to “*n*” specific hosts. For example, a chitinase from *B. thuringiensis* subsp. *pakistani* (GenBank accession number BTU89796) was used to increase the activity of Cry proteins against *Aedes aegypti* (Thamthiankul et al., 2001), and ChiA74 from *B. thuringiensis* subsp. *kenyae* (AF424979) against *S. frugiperda*, *M. sexta*, *P. xylostella* (Lepidoptera), and *Aedes aegypti* (Diptera) (Casique-Arroyo et al., 2007; Barboza-Corona et al., 2014; Juárez-Hernández et al., 2015; González-Ponce et al., 2017). Besides these, chitinases from *B. thuringiensis* subsp. *tenebrionis* strain 9602 (KF671757) were active against *Helicoverpa armigera* (Lepidoptera) and *Caenorhabditis elegans* (nematode) (Ni et al., 2015), whereas *B. thuringiensis* subsp. *tenebrionis* strain DSM2803 (KJ764712) was active against *Colletotrichum gloeosporioides* (fungus) (De la Fuente-Salcido et al., 2016). Based on these observations, most chitinases shows high sequence similarity, but at the same time, it is difficult to assign chitinase specificity to a discrete host group of insects. Our resulting phylogram suggests that chitinases from *B. thuringiensis* form two main groups (I and II, Figure 3). In the first group, chitinases from *B. thuringiensis* subsp. *tenebrionis* with accession numbers KF671757 and KJ764712 are effective against *Helicoverpa armigera*, *Caenorhabditis elegans*, and *Colletotrichum gloeosporioides*, respectively (Ni et al., 2015; De la Fuente-Salcido et al., 2016), but share the same ancestor. Also, *B. thuringiensis* subsp. *pakistani* (U89796) and *B. thuringiensis* subsp. *kenyae* (AF424979) chitinases that are effective against *A. aegypti*, branch in different groups but share a common ancestor (Thamthiankul et al., 2001; Juárez-Hernández et al., 2015). For the second group, there is no report of the chitinase activity against insects, fungi or nematodes. Because there is scarce evidence about chitinases and their target-specific associations, it is evident that more work is needed to establish the evolutionary histories of *B. thuringiensis* chitinases.

Alternatively, *B. thuringiensis* chitinase gene promoters (e.g., *chiA74* and *chiA71*) harbor −35 and −10 consensus sequences that show identity with *E. coli* promoters and also with promoters recognized by the housekeeping/early sporulation σ^A^ factor of *B. subtilis* (Haldenwang, 1995; Thamthiankul et al., 2001; Barboza-Corona et al., 2003; González-Ponce et al., 2019). The fact that chitinase gene promoters from *B. thuringiensis* are recognized by the *E. coli* transcriptional machinery provides an important advantage for heterologous expression because it is relatively much easier to work with *E. coli* than with *B. thuringiensis* for different reasons. For example, *E. coli* has a shorter generation time and is a non-sporogenic bacterium. It has been observed that *B. thuringiensis* synthesizes chitinases at basal levels in media lacking chitin, although synthesis can be induced with chitin and repressed with glucose (catabolic repression), factors which may be irrelevant in *E. coli*. Nevertheless, regardless of whether or not glucose is added to a culture of *B. thuringiensis*, basal chitinase synthesis is not suppressed (Casique-Arroyo et al., 2007; Barboza-Corona et al., 2009; Xie et al., 2010).

The core promoter of the *chiA* gene of *B. thuringiensis* subsp. *israelensis* strain 75 (Bti75) was delimited by fusing the 5′UTR with the β-galactosidase (*bgaB*) gene from *B. stearothermophilus*. When chitin was added to the culture medium, a 2.5-fold increase in the activity of BgaB was observed. Interestingly, a *cis-*active element of ∼16 bp, designed *dre* (DasR responsive elements), is present downstream of the core promoter. The *dre* element plays a role in gene expression as deletion of this sequence resulted in constitutive expression of the chitinase gene (Xie et al., 2012). The *dre* sequence has also been reported in other bacteria, including *Streptomyces* and other *Bacillus* species (Colson et al., 2007; Bertram et al., 2011). In *B. thuringiensis*, *dre* acts as a binding site for a negative transcriptional regulator called YVoABt, an N-acetylglucosamine utilization regulator primarily induced by GlcNAc. YVoABt complexes with the phosphoprotein Hpr-ser_45_-P to bind to *dre* (Jiang et al., 2015) thereby repressing expression of the chitinase gene. However, when the bacterium utilizes chitin as a substrate, GlcNAc or oligosaccharides derived from chitin are produced which leads to the displacement of the repressor and subsequent gene transcription (Jiang et al., 2015; Cao et al., 2018). The YVoABt regulator is also known as NagR_Bt_ because it is an ortholog of NagR in *B. subtilis*. NagRBt is a pleiotropic transcriptional regulator that upregulates and downregulates at least 27 and 14 genes, respectively, in *B. thuringiensis* (Cao et al., 2018).

**FIGURE 4:**
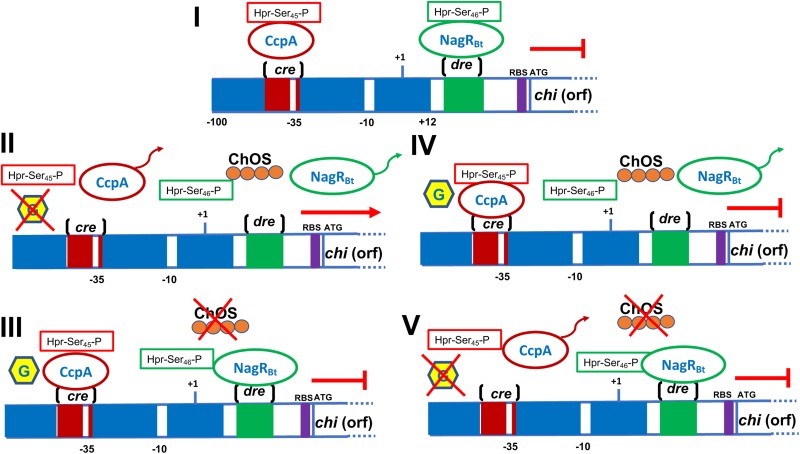
Schematic representation of the *B. thuringiensis* chitinase regulation. **(I)** The *cre* region is localized into the promoter. This region acts as a site binding of the CcpA, regulator involved in catabolic repression which is helped by the Hpr-Ser_45_-P protein in the presence of glucose. Downstream the promoter is found the *dre* sequence, which acts as operator and binding-site for the negative regulator NagR_Bt_. **(II)** When glucose is absent the CcpA regulator is released from the *cre* sequence, the addition of chitin generates inductors (chitin-oligosaccharides, ChOS) that release the regulator NagR_Bt_ from the *dre* sequence. The presence of both inductors (glucose and chitin) allow chitinase transcription. **(III)** When glucose is presents but chitin is absent, CcpA and NagR_Bt_ are bound to the *cre* and *dre* sequences, respectively, blocking the chitinase transcription. **(IV)** Glucose and chitin present, CcpA binds to *cre* sequence whereas NagR_Bt_ is released from *dre*; however, *chi* is not transcribed because of the inhibition through catabolic repression. **(V)** When glucose and chitin are absent, CcpA is released from *cre* but NagR_Bt_ is bound to *dre*, inhibiting the chitinase transcription. This figure was built taking information published in Xie et al. (2012), Deutscher et al. (2006), Jiang et al. (2015), and Cao et al. (2018). Part of a figure published by Jiang et al. (2015) was taken as the basis for the preparation of this figure. Rights and Permissions have been obtained from the American Society for Microbiology and Copyright Clearance Center.

The chitinase gene promoter also contains *cre* (catabolic response element), which is the binding site of a catabolic control protein A (CcpA) that in different Gram-positive bacteria has been identified as the key regulator of the carbon catabolic repression (CCR) process. CcpA can bind specifically to *cre* upon binding to phosphoprotein Hpr-Ser_46_-P (Deutscher et al., 2006). Deletion of the CcpA regulator increases chitinase gene expression and enzyme activity by ∼39-fold (Jiang et al., 2015). Based on previously reports (Deutscher et al., 2006; Xie et al., 2012; Jiang et al., 2015; Cao et al., 2018), we have depicted a model for the regulation mediated by *cre*/CcpA and *dre*/NagR_B_ in Figure 4.

## Application of Chitinases

### To Improve the Toxic Activity Against Insects, Phytopathogenic Fungi and Nematodes

During the 1970s, and 1990s, it was shown that co-application of spore/crystals and heterologous chitinases or spores/crystals and chitinolytic bacteria increased the insecticidal activity of Cry proteins against the spruce budworm (*Choristoneura fumiferana*) and African cotton leafworm (*Spodoptera littoralis*) larvae, respectively (Smirnoff, 1974; Regev et al., 1996). The enhanced toxicity of the Cry proteins was apparently due to degradation of the chitin-based peritrophic membrane of larvae which allowed increased access of the toxins to their respective receptors on the midgut cells (Regev et al., 1996). These observations stimulated the use of heterologous chitinases (i.e., chitinases not synthesized by *B. thuringiensis*), such as those synthesized by *S. marcescens*, *B. circulans*, and *B. licheniformis*, to increase the insecticidal activity of Cry proteins against pest larvae of agricultural importance. For example, ∼0.1 mg/ml chitinase from *S. marcescens* mixed with ∼3 mg per ml of semipurified Cry1C, both produced separately in *E. coli*, caused a maximal reduction in larval weight compared with the use of 20.0 μg of Cry1C per ml without chitinase. Also, the use of a mix of *Pseudomonas fluorescens* transformed with *cry1Ac7* and *chi* of *S. marcescens* showed enhanced toxicity against the sugarcane borer *Eldana saccharina* (Downing et al., 2000). As heterologous chitinases mixed with spores/Cry crystals amplified the toxic effect of Cry proteins (Figure 1), the next step was to transform Bt with heterologous genes coding for chitinases. Wiwat et al. (1996) made the first attempt to transform *B. thuringiensis* subsp. *israelensis* with chitinase genes from *Aeromonas hydrophila* and *Pseudomonas maltophilia*, but expression was rather low. Later, *B. thuringiensis* subsp. *aizawai* was transformed with chitinase genes from *B. circulans*. The recombinant had a higher level of chitinase activity and improvements in toxicity against gypsy moth larvae (*Lymantria dispar*) were observed (Lertcanawanichakul and Wiwat, 2000; Lertcanawanichakul et al., 2004). An interesting observation was that combining 10 mU of *B. licheniformis* chitinases with spores/Cry crystals of *B. thuringiensis* subsp. *aizawai* against *S. exigua* resulted in a decrease in LD_50_ by approximately 15-fold at 7 days, compared to the use of spores/crystals alone. However, when *B. thuringiensis* subsp. *aizawai* was transformed with a chitinase gene from *B. licheniformis*, a low level of chitinase synthesis was observed with no change in the toxic effect when compared with the parental strain (Tantimavanich et al., 1997). Additionally, a chitinase gene from *B. licheniformis* TP-1 was used to transform *B. thuringiensis* subsp. *israelensis*; the transformant produced a lower number of spores and was less toxic to *A. aegypti* larvae when compared with the parental strain (Sirichotpakorn et al., 2001). These observations indicated that the synthesis of heterologous chitinases in *B. thuringiensis* requires optimization of expression systems and/or maintaining the efficacy of the enzyme, which unlike Cry proteins is not crystallized to form a stable inclusion prior to delivery to the larval target. In other regards, whereas most studies focused on the use of chitinases combined with spore/crystal mixtures against lepidopterans and dipterans, specifically mosquito larvae, the potential for such a strategy to control coleopterans was also demonstrated. Okay et al. (2008) showed that an anti-coleopteran recombinant *B. thuringiensis* strain 3023 expressing the *chiA* gene of *S. marcescens* was 5.2- and 1.3-fold more toxic than, respectively, parental 3023 strain and *S. marcescens*.

Because the expression of heterologous chitinases using plasmids can be unstable after a certain number of generations, particularly in the absence of antibiotic selection in the larvae host, the integration of chitinase gene into the bacterial chromosome presented a viable alternative. Thamthiankul et al. (2004) integrated a chitinase gene from *B. licheniformis* under control of the P19 promoter into the chromosome of *B. thuringiensis* subsp. *aizawai* (BTA1) and observed that the growth and sporulation profiles were comparable to the wild-type strain. Preparations of whole culture broth of the recombinant against *S. exigua*, compared with the wild strain, showed a 2.5× reduction in the LC_50_, i.e., from 30.8 microgram/cm^2^ to 12.2 microgram/cm^2^. Years later, the same group transformed BTA1 with the chitinase gene of *B. licheniformis* but under the control of the *cry11Aa* sporulation-dependent promoter. The activity of the recombinant strain against *S. litura* was enhanced by ∼4.6-fold compared to the wild-type strains, a result due to the increase in chitinase synthesis and enzymatic activity which synergized the effect of Cry1A (Buasri and Panbangred, 2012). Besides, endogenous chitinases of *B. thuringiensis* subsp. *israelensis* IPS78 and *B. thuringiensis* subsp. *aizawai* HD133 have also been implicated in the pathogenicity against, respectively, *Culicoides nubeculosus* and *S. littoralis*. This was demonstrated because the use of allosamidin (a chitinase inhibitor) in the bioassays increase the LD_50_ (i.e., less toxic) compared to a control lacking the inhibitor (Sampson and Gooday, 1998), indicating that chitinase inhibition abolishes the contribution of these enzymes to the toxic effects of Cry proteins. It has been shown that wild-type strains of *B. thuringiensis* produce a basal level of chitinase in culture medium, and that synthesis of the enzyme can be induced or repressed with chitin or glucose, respectively (Barboza-Corona et al., 2009; Xie et al., 2010). The levels of chitinase synthesis can be determined by *in vitro* assays, qualitatively by the observation of halo formations in solid medium supplemented with colloidal chitin, or quantitatively by measuring reducing sugars and by fluorescence emission using synthetic fluorogenic chitin-derivates when the bacterium is grown in a liquid medium (Barboza-Corona et al., 1999; Liu et al., 2002; Gómez-Ramírez et al., 2004). As endogenous chitinases of *B. thuringiensis* are involved in virulence against insects (Sampson and Gooday, 1998), secreted chitinases present in a culture medium might be useful to increase the insecticidal activity of *B. thuringiensis*. This was verified when secreted chitinases of *B. thuringiensis* in the culture supernatants when mixed with spores/Cry crystals increased toxicity against *P. xylostella*, *S. exigua*, and *Choristoneura fumiferana* (Wiwat et al., 2000; Liu et al., 2002; Vu et al., 2009).

On the other hand, previous studies revealed that *B. thuringiensis* is antagonistic to phytopathogenic fungi due to the production of lytic enzymes, including chitinases. Therefore, chitinases are also considered as promising biocontrol agents to protect plants, particularly those of high economic value, from infestation and damage by phytopathogenic fungi. The mode of action of *B. thuringiensis* against fungi differs depending on the strains. For example, different morphological effects on fungal cell walls have been noted, such as inhibition of mycelial growth and spore germination, lysis of spores and hyphal tips, and germ tube elongation (Öztopuz et al., 2018). Specifically, the inhibition of phytopathogenic fungi using bacterial suspensions, supernatants, concentrated crude preparations containing chitinases, and purified chitinases of *B. thuringiensis* have been described. It has also been reported that there is no significant difference in antifungal activities of *B. thuringiensis* between the crude extract and purified chitinase (Morales de la Vega et al., 2006), although Melent’ev et al. (2001) showed that purified chitinase was unable to inhibit the growth of *Fusarium oxysporum* and *Helminthosporium sativum* when compared to the activity observed with crude extracts. Isolates of *B. thuringiensis* have shown antifungal potential in dual cultivation assays. For example, Hollensteiner et al. (2017) showed *in vitro* antagonism toward *Verticillium dahlia*, a pathogen with a broad host range, including economically important crops such as tomato. The authors suggested that the inhibitory effect on the growth of the phytopathogens was attributed to the presence of chitinases in the supernatant of the bacterial culture. Similar results were obtained with supernatant preparations against *Aspergillus niger*, *A. foetidus*, and *A. ochraceus* (Driss et al., 2005; Öztopuz et al., 2018) in which the antifungal effect was attributed not only to the presence of chitinases but also to a complex of hydrolytic enzymes, including proteases and gluconases. Regardless, inhibitory effects against fungi have been demonstrated using partially purified or purified chitinases of *B. thuringiensis*, and these species-specific enzymes were more effective than those produced by *B. licheniformis* against species of *Fusarium*, *Rhizopus*, and *Trichoderma* (Morales de la Vega et al., 2006; Gomaa, 2012; Mehmood et al., 2015). More recently, a purified chitinase from *B. thuringiensis* subsp. *tenebrionis* DSM-2803 was shown to adversely affect the growth of *Colletotrichum gloeosporioides*, a potent phytopathogen that causes “anthracnose” in avocado (*Persea americana*), and one that is responsible for significant economic loss in México (De la Fuente-Salcido et al., 2016). Other studies have also shown the adverse effect of *B. thuringiensis* chitinases against *F. oxysporum*, the etiological agent of the fungal vascular wilt of date palm, an important traditional crop in North Africa and The Middle East (Ni et al., 2015; Djenane et al., 2017). Phytopathogenic fungi alter plant physiology and cause disease throughout development, including during seed germination. Therefore, the effect of chitinase on germination of seeds infested with phytopathogenic fungi has also been evaluated. Gomaa (2012) showed that crude extracts from *B. thuringiensis* increased the rate of germination of soybean seeds infested with phytopathogens (e.g., *Aspergillus terreus*) by 7.6-fold when compared to controls. Similar positive effects were observed by Morales de la Vega et al. (2006) with the treatment of seeds infested with *Pestalotia* sp. and *A. niger*. Furthermore, Seo et al. (2012) reported that chitinase produced by *B. thuringiensis* GS1 inhibits the growth of *Rhizoctonia solani* in cucumber plants. Finally, it is also interesting to note that chitinases can induce plant defenses against these microbial pathogens. For example, when rice seedlings were treated with immobilized purified chitinase from *B. thuringiensis* H3, an increase in the synthesis of defense enzymes, including peroxidase and phenylalanine ammonia-lyase, was observed (Tang et al., 2012).

While several studies have demonstrated the potential use of *B. thuringiensis* chitinase to control insects and fungi, only a few have been reported on the effect of these enzymes on free-living nematodes such as *Caenorhabditis elegans.* Studies by Zhang et al. (2014) suggested that the detrimental activity of *B. thuringiensis* strain 010 against *C. elegans* could be due to the action of chitinases. More recently, a mutant (ChiW50A) of chitinase Chi9602 of *B. thuringiensis* subsp. *tenebrionis* YBT-9602 with ∼60% higher enzymatic activity elicited an increase of ∼20% in mortality against *C. elegans* when compared with the parental strain (Ni et al., 2015). Moreover, nanoparticles made of immobilized *B. thuringiensis* chitinases (i.e., Chi9602) showed enhanced nematicidal effects against *C. elegans* (Qin et al., 2016).

### To Generate Antibacterial Chitin-Derived Oligosaccharides

Chitinases of *B. thuringiensis* subsp. *pakistani* (ChiA71), *B. thuringiensis* (Chi255) and *B. thuringiensis* subsp. *aizawai* have been shown to hydrolyze colloidal chitin to primarily produce GlcNAc and (GlcNAc)_2_ suggesting that these enzymes possess an exo-type chitinase activity (Thamthiankul et al., 2001; Driss et al., 2005; Morales de la Vega et al., 2006). In particular, thin layer chromatography (TLC) demonstrated that ChiA74 of *B. thuringiensis* subsp. *kenyae* when produced in *E. coli* can generate chitin-derived oligosaccharides (ChOGs) with degrees of polymerization higher than 2, suggesting an endochitinase activity. However, more recently it was shown that purified ChiA74 has a processive activity generating mainly GlcNAc and (GlcNAc)_2_ when using colloidal chitin as substrate, suggesting that purified ChiA74 has an exochitinase action and the previous endochitinase activity could be erroneous because the ChiA74 preparation could have been mixed with other enzymes in crude extracts (Casados-Vazquez et al., 2015; Juárez-Hernández et al., 2019). Nevertheless, the resulting ChOGs exhibited antimicrobial activities against a number of clinically significant Gram-positive and Gram-negative bacteria, including *B. cereus*, *Listeria monocytogenes*, *Staphylococcus aureus*, *Staphylococcus xylosus*, *Klebsiella pneumoniae*, *Pseudomonas aeruginosa*, *Shigella sonnei*, *Shigella flexneri*, and *Proteus vulgaris* (Barboza-Corona et al., 2008; Ortiz-Rodríguez et al., 2010; Castañeda-Ramírez et al., 2013).

## Development of Recombinant *B. thuringiensis* Strains Expressing Homologous Chitinase Genes

Chitinase genes from *B. thuringiensis* have been used as a resource to generate recombinant strains of the same species. These recombinants can be classified into three categories: (i) strains expressing chitinases that can be secreted and collected from supernatants, (ii) recombinants that synthesize chitinases lacking the secretion signal thereby leading to amorphous intracellular inclusions of the enzyme that are released together with the spores/crystals following autolysis, and (iii) strains producing inclusions composed of the chitinase fused in frame with the C-terminal (crystallization) domain of Cry proteins (Figure 1). Currently, there is no report about the development of *B. thuringiensis* strains with homologous chitinase genes inserted into the chromosome.

Regarding the first category, *B. thuringiensis* HD73 was transformed with chitinase *chiA74* under the control of the wild-type promoter (HD-73-pEHchiA74) or the *pcytA-STAB* system (HD-73-pEBchiA74) (Casique-Arroyo et al., 2007; Barboza-Corona et al., 2009). The *pcytA* sequence is composed of three strong sporulation-dependent promoters [BtI(σ^E^), BtII(σ^K^), BtIII(σ^E^)] which contribute to the high expression level, and the STAB sequence contributes to the stabilization of the mRNA (Park et al., 1998; Sakano et al., 2017). HD-73-pEBchiA74 showed an increase in enzymatic activity of 58- and 362-fold higher than HD-73-pEHchiA74 and parental HD-73, respectively, but with a compensating reduction in Cry crystal size and number of viable spores when compared to the wild-type HD73. This, unfortunately, did not increase in toxicity against *P. xylostella* when compared to the parental strain (Casique-Arroyo et al., 2007).

Concerning the second category, the deletion of the secretion signal peptide led to intracellular accumulation and formation of inclusion bodies composed of the enzyme (Barboza-Corona et al., 2014). The premise for developing *B. thuringiensis* strains that synthesize chitinase as intracellular inclusions is based on the fact the upon autolysis crystals, spores and chitinase inclusion bodies are released simultaneously. For example, a chitinase from *B. thuringiensis* strain 4.0718 under the control of dual overlapping promoters plus Shine-Dalgarno sequence and terminator sequence of *cry1Ac3* was introduced into Cry^–^B, an acrystalliferous *B. thuringiensis* strain that does not produce Cry crystals. Chitinase inclusion bodies were formed and exhibited chitinase activity. When these inclusion bodies were mixed with Cry crystals, an increase in the toxicity of Cry1Ac against larvae of *S. exigua* and *Helicoverpa armigera* was observed (Hu et al., 2009). As the authors transformed an acrystalliferous strain, they could not study the effect on the production of the insecticidal crystal proteins. To produce chitinase inclusion bodies and Cry crystals in the same *B. thuringiensis* host, *chiA74*Δsp lacking the signal peptide sequence under the control of the *pchiA* wild-type promoter (pEHchiA74Δsp) or the *pctyA*-STAB system (pEBchiA74Δsp) was introduced into *B. thuringiensis kurstaki* HD1 and *B. thuringiensis* subsp. *israelensis* IPS-82, strains that are commonly used in formulations of commercial products. In HD1 ChiA74Δsp inclusions were dissolved in alkali and reducing conditions, similar to Cry crystals, and the enzyme retained its activity in a wide range of pH (5–9) but showed a drastic reduction (∼70%) at pH 10. Compared to parental HD1 strain, the recombinant HD1-pEBchiA74Δsp showed a 42-fold increase in chitinase activity and a 3-fold increase in the number of viable spores, but the bipyramidal Cry crystals were ∼30% smaller. Bioassays against first instar larvae of *M. sexta* with spore-crystals of HD1 or spore-crystal-ChiA74Δsp inclusions of HD1-pEBchiA74Δsp showed LC_50_ of 67.30 and 41.45 ng/cm^2^, respectively (Barboza-Corona et al., 2014). When *B. thuringiensis* subsp. *israelensis* was transformed with *chiA74Δsp*, the recombinants produced their native Cry crystals composed of Cry4Aa, Cry4Ba, Cry11Aa, and Cyt1Aa and also ChiA74Δsp inclusions. These recombinants showed ∼3-fold increase in both chitinase activity and viable spore count when compared with the parental strain and were twofold more toxic than *B. thuringiensis* subsp. *israelensis* against fourth instars of *A. aegypti* larvae (Juárez-Hernández et al., 2015).

Finally, chimeric fusions of chitinase with the C-terminal domains of Cry proteins have been reported in two studies. In the first study, the sequence coding a chitinase of *B. thuringiensis* subsp. *kurstaki* BUPM255 under the regulation of pBtI-BtII was transcriptionally fused to the C-terminal domain of *cry1Ac* and the construct was used to transform *B. thuringiensis* BNS33. The recombinant showed a 2.5-fold increase in chitinase activity and was 1.5× more toxic to *Ephestia kuehniella* when compared to the parental strain (Driss et al., 2011). Unfortunately, the authors did not report whether the chimeric protein formed inclusions or retained enzymatic activity in lyophilized preparations. A similar study was performed by González-Ponce et al. (2017), where the *chiA74* gene lacking its secretion signal peptide sequence (*chiA74Δsp*) was fused in frame with the sequence coding for the C-terminal crystallization domain and transcription terminator of *cry1Ac*, under the regulation of s*pcytA-p/*STAB-SD promoter system, and the construct was used to transform *B. thuringiensis* subsp. *kurstaki* HD73. Amorphous inclusions apparently composed of the chimera and native bipyramidal Cry1Ac crystals were observed. The chitinase activity of purified Cry1Ac/amorphous inclusions was 51-fold higher when compared to purified Cry1Ac inclusions of parental HD73. Bioassays against larvae of *S. frugiperda* with spore/crystals of HD73 or spore-crystals-ChiA74Δsp chimeric inclusions of recombinant HD73 showed a reduction of 30% in the LC_50__’__s_ when compared to wild-type HD73, indicating that the enhancement in toxicity directly correlated to the presence of the atypical amorphous component.

## Future Perspective

*Bacillus thuringiensis* is a fascinating microbe for several reasons, but primarily for its ability to produce a plethora of molecules that have applied or potential applied value. Unequivocally, this microbe should be viewed as a microbial factory that can be engineered and exploited for simultaneous production of Cry and Cyt proteins, VIPs, parasporins, chitinases, and other metabolites that target vectors of human diseases, cancers, clinically significant microbial pathogens, and agricultural pest.

In spite of recent advances in the study of the *B. thuringiensis* chitinases, several issues need to be resolved. In particular, with regard to its entomocidal potential, it has been demonstrated that chitinases from *B. thuringiensis* increase the toxic effect of Cry proteins, but there is no evidence to show whether these enzymes act synergistically or merely potentiate the activity of Cry. There appears to be little or no evidence to support a hypothesis that substantial increases in chitinase production and enzymatic activity translate to substantial decreases in LC_50__’__s_. In this regard, definitive studies are required to determine the fate of chitinases in the insect midgut after ingestion. Are these enzymes relatively stable or are they rapidly degraded by digestive proteases? Indeed, it is not clear whether the chitinolytic activity of a particular chitinase is insect-species specific, or whether optimal catalysis for the enzyme is dependent on the organization and variation of chitin in the peritrophic membrane. Our phylogenetic analysis suggests that chitinases from *B. thuringiensis* can be grouped in different clusters, but whether members in these clusters have species-specific targets requires an extensive study that realistically will not be completed in the near future. Assuming that chitinases are stable and active in the physical and biochemical environment of the midgut, at least for the time required for activation of Cry toxins, the questions of whether inclusions of the enzyme produced in microbial factories (including *B. thuringiensis*) dissolve efficiently *in vivo*, and if the solubilized forms of the enzyme refold properly for effective catalysis are interesting and worth addressing. The extent of antifungal and nematicidal effects of chitinases produced by *B. thuringiensis* remains questionable in our view. More focused studies on a variety of these enzymes assayed on individual fungal and nematode species could provide valuable insights into selecting the best, or suitable, candidates for further research and development. Moreover, basic molecular research is required to elucidate the translocation mechanism(s) of chitinases in *B. thuringiensis*, although it is likely that a system analogous to that in *E. coli* is involved, based on the observation that chitinases of *B. thuringiensis* origin are secreted by this Gram-negative bacterium. Moreover, whether the chitinases of *B. thuringiensis* have a processive or non-processive mechanism of chitin hydrolysis needs to be determined.

Finally, due to the structural differences and characteristics that chitinases have, their use in specific practical applications can be difficult to determine and optimize (Oyeleye and Normi, 2018). However, we believe that many of these issues will ultimately be resolved and that the recent structural analyses (Packer and Liu, 2015; Juárez-Hernández et al., 2019) will be indispensable toward this end. With this knowledge, and with the use of genome editing technologies and new synthetic biology and computational tools, the performance of chitinases could be improved. For instance, due to the modularity of chitinases, the creation of new synthetic chitinases derived from a combination of different protein domains could enable the creation of new tailor-made chitinases with new functions. These new synthetic chitinases could then be used for specific biotechnological applications and potentially help increase the market demand for chitinolytic enzymes derived from microbial cell factories like *B. thuringiensis*.

## Author Contributions

SM-Z, UB-C, GH-G, DB, and JB-C wrote the manuscript and making substantial contributions to the work. All authors read and approved the final version of the manuscript.

## Conflict of Interest

The authors declare that the research was conducted in the absence of any commercial or financial relationships that could be construed as a potential conflict of interest.

## Footnotes

1 http://www.cazy.org/Glycoside-Hydrolases.html

2 www.ncbi.nlm.nih.gov/

3 www.ebi.ac.uk/interpro/beta/

4 https://www.ncbi.nlm.nih.gov/

5 https://www.ebi.ac.uk/Tools/msa/muscle/

6 https://www.hiv.lanl.gov/content/sequence/findmodel/findmodel.html
